# Transcriptomic Analysis and Physiological Indicators Synthetically Uncover Candidate Genes Regulating Polyembryony Formation of *Phoebe bournei* Seeds

**DOI:** 10.3390/biology15120919

**Published:** 2026-06-12

**Authors:** Guoxin Mei, Lulu Wan, Zeping Jian, Baiyou Chen, Xiaowen Li, Bao Liu, Jun Xiong, Shipin Chen, Haichao Hu

**Affiliations:** 1 College of Forestry, Fujian Agriculture and Forestry University, Fuzhou 350002, China; 18291693423@163.com (G.M.); jianzeping1015@163.com (Z.J.); 18106969263@163.com (B.C.); 12404021007@fafu.edu.cn (X.L.); fafulb@163.com (B.L.); 2 Fuqing Municipal Bureau of Natural Resources and Planning, Fuqing 350399, China; wan201920@163.com; 3 College of Life Science, Fujian Agriculture and Forestry University, Fuzhou 350002, China; xj_x0347@163.com

**Keywords:** *Phoebe bournei*, polyembryony, transcriptome, RWP transcription factor, seed development

## Abstract

*Phoebe bournei* is a precious timber species unique to China, possessing significant ornamental, ecological, and medicinal values. The polyembryonic phenomenon is widely observed in its seeds, but the underlying mechanisms driving its formation remain unclear. In this study, an integrated analysis was conducted for polyembryony formation in *P. bournei* seeds, including biochemical indicators, environmental factors, and transcriptional levels. Firstly, morphological observation of seeds from 13 plus trees showed no significant correlation between polyembryony rate and external environment or seed morphology. Subsequently, comparative transcriptomic analysis between monoembryonic and polyembryonic seeds identified 1957 differentially expressed genes (DEGs), which were significantly enriched in biological processes and pathways including photosystem II, tetrapyrrole binding, heme binding, and phenylpropanoid biosynthesis, indicating the probable effect of gene regulation in polyembryony formation. Furthermore, eight RWP transcription factors were identified, potentially involved in polyembryony. qRT-PCR analysis revealed that the expression levels of *PbRWP2* and *PbRWP5* were significantly upregulated in polyembryonic seeds, indicating they may be key regulatory genes during polyembryony formation in *P. bournei* seeds. This study preliminarily explored the transcriptomic characterization and potential molecular regulatory basis of polyembryony formation in *P. bournei*, laying a critical foundation for further deciphering the intricate mechanisms governing its polyembryonic development.

## Simple Summary

*Phoebe bournei* is a unique and precious tree species in China with multiple values. Its seeds often exhibit polyembryony, where multiple embryos develop within a single seed, but the underlying cause of this phenomenon remains unclear. This study aimed to explore the mechanism of polyembryony in *P. bournei* seeds by analyzing three aspects: seed biochemical characteristics, growth environment, and gene expression. Our findings indicate that environmental conditions and seed morphology are not significantly correlated with polyembryony, whereas differences in gene expression may be the key factor. Furthermore, two specific genes were found to exhibit higher expression levels in polyembryonic seeds, suggesting they may be involved in regulating polyembryony formation. This study provides a reference for a deeper understanding of the polyembryonic development mechanism in *P. bournei* and holds great significance for the cultivation and conservation of this precious tree species.

## 1. Introduction

*Phoebe bournei*, an evergreen broad-leaved arbor species in the *Lauraceae* family, is an endemic species under Class II national key protection in China. Valued for outstanding economic, ecological, medicinal and ornamental attributes, *P. bournei* constitutes an elite precious timber resource. Featuring straight trunks, dense wood texture, excellent durability and unique fragrance, the species is commonly named “golden-thread nanmu” and extensively exploited for shipbuilding, architectural engineering and premium artistic carving [1]. Its primary suitable distribution areas cover subtropical regions in China, including provinces Zhejiang, Jiangxi, Fujian, Guangdong, Guangxi, Hunan, Hubei, Guizhou, and Chongqing, while its natural distribution tends to be scattered and fragmented [2]. Overexploitation driven by anthropogenic activities in recent decades has drastically depleted its natural germplasm reserves. Accordingly, artificial cultivation has become the dominant approach for species conservation and sustainable resource exploitation [3]. Given the immature status of current asexual propagation protocols, seed-based seedling production still dominates practical forestry cultivation. Against this background, systematic research on seed biological traits is essential to facilitate germplasm preservation and genetic improvement programs for *P. bournei*.

In angiosperms, the seed embryo originates from the fusion of egg cells and sperm during sexual reproduction. Usually, plant seeds contain only one embryo that develops after fertilization, also called a zygotic embryo. Polyembryony describes the occurrence of two or more embryos within a single seed [4]. The formation mechanisms of polyembryos mainly include four types [5]. The first type is zygotic cleavage embryony, which refers to the division of the fertilized egg resulting in the formation of multiple independent embryos, which is common in gymnosperms. The second type is asexual gametic polyembryology, formed by the development of non-embryonic cells such as polar nuclei, parietal cells, or antipodal cells. The third type is sporophyte-free polyembryology, which involves the direct development of nucellus, integument, and microstipe into embryos. The last type is called pseudo-polyembryology, resulting from the production of multiple embryo sacs within the same ovule. The vast majority of polyembryos originate from apomixis, including agametic and aposporous reproduction [6]. As an asexual reproductive strategy, apomixis produces embryos from ovule tissues without meiosis or fertilization, yielding progeny genetically identical to the maternal plant and thus showing great potential for plant breeding [7,8]. Given the considerable conservation and breeding demands of *P. bournei*, investigating its seed polyembryony is therefore of important practical value.

The phenomenon of polyembryony was first observed in plants by Leeuwenhok in *Citrus sinensis*, where each seed was found to contain two embryos capable of developing into two independent seedlings [6]. In the polyembryonic maize, a higher frequency of polyembryony has been associated with increased phytochemical content and improved seed germination [9]. Moreover, polyembryonic maize has higher growth, moisture, protein content, technical functional characteristics, and antioxidant activity than monoembryonic counterparts [10]. Metabolite analysis of mango polyembryonic genotype ovaries shows that the formation of multiple embryos is closely related to high concentrations of plant hormones such as indole-3-acetic acid (IAA), indole-3-butyric acid (IBA), and salicylic acid (SA) [11]. At the molecular level, RWP-RK is a highly conserved transcription factor family in plants, primarily regulating the differentiation of plant reproductive cells and early embryonic morphogenesis [12]. Phylogenetic analyses have classified the plant RWP-RK family into two subfamilies: NLP and RKD. The NLP subfamily functions in response to nitrogen signals, while the RKD subfamily specifically modulates the development of female gametophytes, embryo sac formation and apomixis. This functional divergence is highly conserved among terrestrial plants [13]. Experimental studies on model plants further clarify its embryonic regulatory function. In *Arabidopsis thaliana*, RKD4 regulates zygote activation and the early embryonic morphogenesis, and gene mutations lead to abnormal embryonic development [14]; *AtRKD5* can inhibit autopolyploid reproduction mediated by the BBM gene and precisely regulate the process of autonomous embryo formation in plants [15]. Genomic investigations on woody plants and crops have also verified the conserved functions of this gene family. In *Zanthoxylum armatum*, genes of the RKD subfamily are specifically expressed in reproductive organs and are strongly associated with the polyembryony trait derived from apomixis [16]; The *MiRWP* gene in mango modulates polyembryony and exhibits convergent evolution with its homologs in citrus. The developed KASP and PACE molecular markers provide technical support for apomixis-related breeding [17]; RKD genes in rice are specifically involved in ovule and female gametophyte development, further confirming the cross-species conservation of their regulatory functions in plant reproduction [18]. In addition, *FhRWP* in citrus can directly induce the formation of anomalous embryos in the nucellus, and is the core regulatory gene for polyembryony formation in fruit trees [19]. Currently, studies on the mechanism of polyembryony formation have been completed in various plants such as *Santalum album* [20], *Handroanthus chrysotricus* [21], and *Zea mays* [9]. Although there is a foundation for the research on plant polyembryony, there are still many gaps that need to be filled. The current research mainly focuses on annual crops and Rutaceae fruit trees, while the research on the polyembryony regulatory mechanism of slow-growing and precious broad-leaved tree species of the *Lauraceae* family is very weak; at the same time, the mechanism underlying polyembryony in *P. bournei* remains unclear.

Given this, the present study was preliminarily conducted to explore the morphological characteristics of polyembryonic seeds of *P. bournei* and analyzed the potential factors of polyembryology formation with two scientific hypotheses: (1) intrinsic molecular mechanisms may affect the key factors of polyembryony; (2) transcription factors play an important role in regulating the process of polyembryony. Based on the genome data of *P. bournei* [22], a comparative transcriptome analysis of monoembryonic and polyembryonic seeds was carried out. Relevant genes related to polyembryonic morphogenesis in *P. bournei* were screened, and the expression levels of key genes were verified by quantitative reverse transcription polymerase chain reaction (qRT-PCR). This result provided a reference for the cultivation of *P. bournei* seedlings and laid the foundation for in-depth research on the molecular mechanism of polyembryonic regulation in *P. bournei* seeds.

## 2. Materials and Methods

### 2.1. Description of Plant Material and Sampling Sites

**Figure 1:**
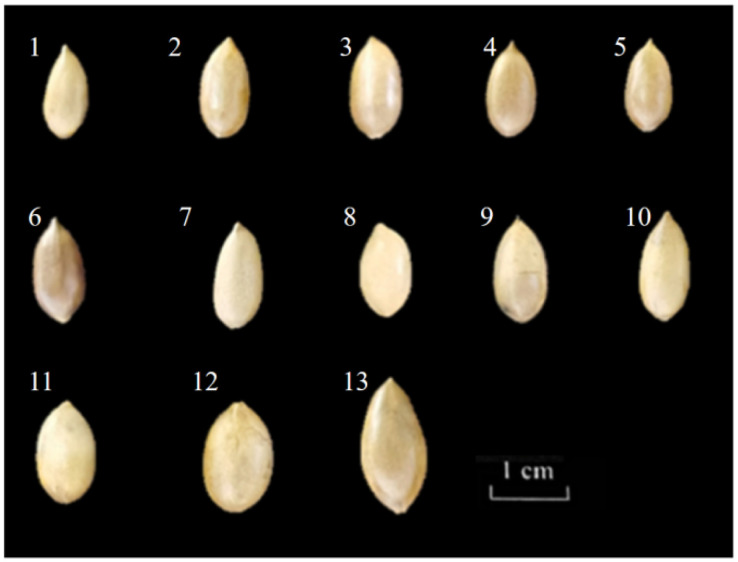
Morphological characteristics of seeds of 13 plus trees in *P. bournei*.

The *P. bournei* seeds used in this study were collected in December 2023 from 13 plus trees (numbered 1 to 13) in Fujian Province. The selected plus trees are strong and healthy, free from obvious pests and diseases, over 25 years old, bear fruit stably, and are at a considerable distance from each other. During collection, the geographical coordinates (including longitude, latitude, and altitude) and morphological indicators (including tree height, diameter at breast height, height to the first branch, and crown width) of each mother tree were systematically recorded. The specific data are shown in Table 1. The fruits of all plus trees were collected simultaneously. After manual peeling, the fruits were rinsed clean with water to obtain pure seeds. The morphological characteristics of the seeds are shown in Figure 1.

### 2.2. Polyembryony Identification and Seed Treatment

#### 2.2.1. Observation of Morphology in Polyembryonic Seeds

In order to explore the polyembryonic morphological characteristics of *P. bournei* seeds, 50 *P. bournei* fruits with complete appearance and well developed were randomly selected from each of the 13 plus trees for dissection. Under a stereomicroscope (Nikon Corporation, Tokyo, Japan), the morphology, number, and position of the embryos within the seeds were observed and counted, and photos were taken for records. This was to clarify the internal structural differences between monoembryonic and polyembryonic seeds.

#### 2.2.2. Polyembryony Rate Statistics

Based on the above anatomical observations, the number of polyembryonic seeds in each plus tree was counted, and the polyembryony rate was calculated. The calculation formula is as follows:

Polyembryony Rate (%)=(Polyembryonic seeds/Total seeds)×100%

#### 2.2.3. Thousand Grain Weight Measurement

Seeds of *P. bournei* with plump, uniform size, intact appearance and no pests or diseases were selected from each plus tree. Three biological replicates were set for each tree, and 100 intact seeds were randomly chosen for each replicate. The weight of seeds per replicate was measured using an electronic analytical balance (readability: 0.0001 g, Mettler Toledo, Greifensee, Switzerland), and the thousand grain weight was calculated accordingly. All results were presented as the mean ± standard deviation.

#### 2.2.4. Seed Rooting Treatment

In the root observation assay, 50 sound, undamaged seeds without pests or diseases were sampled per plus tree, resulting in a total of 650 seeds from 13 plus trees. Selected *Phoebe bournei* seeds were first surface-sterilized via immersion in 0.3% carbendazim solution for 2 h. Following surface disinfection, the sterilized seeds were rinsed three times using purified water and subsequently subjected to a 6 h soaking treatment in a thermostatted water bath maintained at 45 °C. Afterwards, the seeds were sequentially immersed in diluted plant growth regulator solutions: 2 mL of 3% gibberellin emulsifiable concentrate and 3 mL of 5% naphthalene acetic acid aqueous solution were individually diluted with 1 L purified water, and seeds were soaked in each prepared solution for one full day separately. After the two sequential soaking treatments, seeds were rinsed three more times with purified water and arranged on moist paper beds for germination tests. Germination incubation was conducted in 11-cm-diameter Petri dishes lined with wet absorbent cotton overlaid with a sheet of filter paper. The seeds were cultured in a light incubator at 26 °C for 12 h for a total of 22 days. During this period, the paper bed was periodically sprayed with purified water, and seed rooting was observed and recorded regularly.

### 2.3. Transcriptome Sequencing Sample Collection

**Figure 2:**
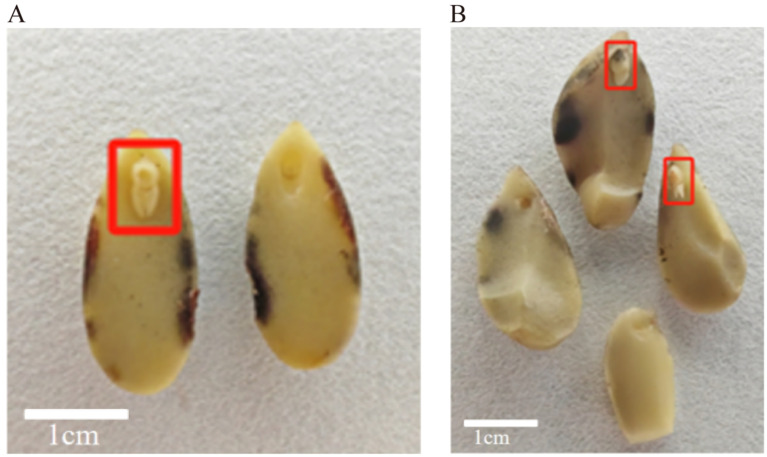
*P. bournei* seed sampling sections. (**A**) Sampling part of monoembryonic seeds; (**B**) Sampling part of polyembryonic seeds.

Transcriptome sequencing was performed on monoembryonic and polyembryonic seeds derived from the same *P. bournei* clone (Figure 2A,B). The instruments were sterilized. Embryos were separated from the seeds using a scalpel. These samples were rinsed three times with purified water, and the surface moisture was absorbed using sterile filter paper. After processing, the embryo samples were placed in cryotubes and rapidly immersed in liquid nitrogen for cryopreservation, then transferred to an ultra-low temperature freezer at −80 °C to maintain RNA integrity for subsequent extraction. Two comparison groups were set up: monoembryonic (Me) seeds (control group) and polyembryonic (Pe) seeds (treatment group). Each group was set up with three biological replicates, resulting in a total of 6 samples. All the frozen samples were sent to Beijing Novogene Bioinformatics Technology Co., Ltd. (Beijing, China) for transcriptome sequencing in January 2024.

### 2.4. Transcriptome Sequencing and Analysis

#### 2.4.1. RNA Extraction, Library Construction, and Sequencing

Transcriptome sequencing of *P. bournei* monoembryonic and polyembryonic samples was performed by Beijing Novogene Bioinformatics Technology Co., Ltd. Total RNA was extracted from monoembryonic and polyembryonic embryos using a plant RNA purification kit (Invitrogen, Carlsbad, CA, USA). The extraction procedure strictly followed the kit’s instructions to avoid RNase contamination, sample degradation, and interference from residual polysaccharides and polyphenols, thereby preserving RNA integrity and purity. RNA integrity was verified by agarose gel electrophoresis, and RNA purity and concentration were measured using spectrophotometry. Only high-quality RNA samples free of degradation and contamination were used for downstream experiments. Qualified total RNA was employed to construct cDNA libraries with the Illumina TruSeq RNA Library Preparation Kit (Illumina, San Diego, CA, USA). Library quality was validated, and paired-end sequencing was conducted on the Illumina NovaSeq 6000 platform (Illumina, San Diego, CA, USA). Three biological replicates were established for each embryo type; total RNA was extracted independently, and libraries were constructed separately for each replicate. Sequencing data quality was assessed using FastQC v0.11.9 and Trimmomatic v0.39 software.

#### 2.4.2. Sequencing Data Quality Control and Comparison

The raw sequencing data (raw reads) were subjected to quality control filtering after sequencing, removing reads containing sequencing adapter sequences, unknown base N, and low-quality reads (reads with a base number of Qphred ≤ 20 accounting for more than 50% of the entire read length) to obtain high-quality effective data (clean reads). Meanwhile, the Q20, Q30, and GC content of the clean reads were calculated to ensure data quality. The assembly and annotation data of the *P. bournei* genome used for comparative analysis were downloaded from the National Genomics Data Center (NGDC, https://ngdc.cncb.ac.cn/ (accessed on 18 June 2025)). Based on the publicly available *P. bournei* whole genome sequence, high-quality clean reads were aligned to the reference genome using HISAT2 v2.0.5, to determine their precise genomic locations.

#### 2.4.3. Gene Expression Quantification and Differential Expression Analysis

Based on the genomic alignment results of clean reads, featureCounts v1.5.0 was used to calculate the raw counts of each gene. These raw counts were not pre-normalized and were directly applied for subsequent differential expression analysis. Meanwhile, Fragments Per Kilobase of transcript per Million mapped reads (FPKM) values were calculated from the raw counts merely for visualization of gene expression levels. Differentially expressed genes (DEGs) were identified using the R package DESeq2 v1.38.3 with a negative binomial distribution model. Genes with an adjusted *p* value (*p*_adj_) < 0.05 and |log_2_Fold Change| > 1 were defined as significant DEGs. We summarized the number of all up- and downregulated expressed genes and analyzed their overall distribution. Hierarchical clustering was performed on the FPKM values of all DEGs to explore their expression patterns across the two groups and three biological replicates. Venn diagrams were used to identify common and specific DEGs among three biological replicates and screen stably expressed core DEGs. Volcano plots were generated to visualize the overall distribution of up- and downregulated DEGs.

#### 2.4.4. Functional Enrichment Analysis of DEGs

To explore the biological functions of DEGs, this study used the corrected *p* value (*p*_adj_) < 0.05 as the threshold for significant enrichment. The screened DEGs were systematically annotated against the GO (Gene Ontology, http://geneontology.org/ (accessed on 7 July 2025)) and KEGG (Kyoto Encyclopedia of Genes and Genomes, http://www.genome.jp/kegg/ (accessed on 7 July 2025)) databases. GO term enrichment and KEGG pathway enrichment were implemented via the R package clusterProfiler v3.8.1 to analyze the biological processes, cellular components, molecular functions, and related metabolic pathways involved in these DEGs. Meanwhile, the number of DEGs annotated to different functional categories was statistically summarized, and a transcription factor distribution map of DEGs was constructed.

### 2.5. RWP Gene Family Identification and Bioinformatics Analysis

#### 2.5.1. Identification of RWP Gene Family Members in *P. bournei* Genome

To identify the RWP gene family members in the *P. bournei* genome, the amino acid sequence of the RWP protein from *Arabidopsis thaliana* (https://www.arabidopsis.org/ (accessed on 18 July 2025)) was used as a probe for local BLAST search (TBtools-II v2.482 E-value < 1 × 10^−3^), and then the obtained sequences were subjected to Blastp reverse search using National Center for Biotechnology Information (NCBI). At the same time, the HMM model of the RWP conserved domain (PF02042) was downloaded from the Pfam database (http://pfam.xfam.org/ (accessed on 18 July 2025)), and the RWP protein was retrieved using Hmmer v3.3.2 [23] software. The protein sequences identified by both methods were merged, and the conserved domains of the candidate proteins were confirmed using online tools such as the CDD database (Conserved Domain Database) of the NCBI, (https://www.ncbi.nlm.nih.gov/Structure/cdd/wrpsb.cgi/ (accessed on 18 July 2025)), the Pfam database, and InterPro (https://www.ebi.ac.uk/interpro/search/sequence/ (accessed on 19 July 2025)). We used the online ExPASy tool (https://www.expasy.org/ (accessed on 19 July 2025)) to characterize the physicochemical properties of the identified proteins.

#### 2.5.2. Phylogenetic Analysis and Sequence Alignment

To study the evolutionary relationship of *P. bournei* RWP proteins, the rice RWP protein sequence file was downloaded from the RICE database (http://rice.uga.edu/ (accessed on 20 July 2025)); the soybean RWP protein sequence (https://www.mdpi.com/article/10.3390/genes14020369/s1/ (accessed on 20 July 2025)) was obtained from the supplementary material of the published literature [24], and was renamed in the order in which it was downloaded. Multiple sequence alignment of the RWP protein sequences of *P. bournei*, rice and soybean was performed using the default parameters of the Clustal X program in the MEGA X [25]. A phylogenetic tree was constructed using the Neighbor-joining (NJ) method to determine the key subfamily branches, and 1000 bootstrap replications were conducted to evaluate the statistical support of the branches.

#### 2.5.3. Gene Expression Pattern and qRT-PCR Verification

Based on the transcriptome sequencing results, the FPKM expression levels of the RWP gene in *P. bournei* were extracted. After log_2_ transformation of the FPKM values, a gene expression heatmap was plotted using the TBtools-II v2.482 [26] software to visually display the expression pattern of the RWP gene in different embryogenic materials.

To verify the reliability of the transcriptome data and analyze the expression of the RWP gene, candidate reference genes were selected. The steps of qRT-PCR were carried out according to the instructions of the kit (Taq Pro Universal SYBR Qpcr Master MIX, Vazyme Biotech Co., Ltd., Nanjing, China), and the reactions were conducted on the Roche LightCycler 480 II real-time fluorescence quantitative PCR instrument (Roche Diagnostics GmbH, Mannheim, Germany). The reference gene used was G1 (Maker00008776), and the reaction system and program as well as the primer sequences used are shown in Supplementary Tables S1 and S2.

### 2.6. Data Processing and Statistical Analysis

In this study, all physiological indicators and quantitative experiments of gene expression were set with 3 biological replicates and 3 technical replicates. Raw data were organized using Microsoft Excel 2021, and the charts were drawn by combining the use of TBtools-II v2.482 and the Novogene Cloud Platform (https://magic-plus.novogene.com/#/login/ (accessed on 27 July 2025)). The phenotypic data (polyembryony rate, thousand-grain weight, morphological indicators) were analyzed using SPSS 26.0 software (IBM Corp., 2019, (accessed on 12 January 2025)). The differences between the two groups were tested using the independent sample *t*-test, and the differences among multiple groups were analyzed using one-way ANOVA. Multiple comparisons were performed using the Duncan method, with the significance level set at *p* < 0.01. The qRT-PCR data were calculated as 2^−ΔΔCt^ for relative expression levels. The differences between groups were tested using the *t*-test, and the results were expressed as mean ± standard deviation.

## 3. Results

### 3.1. Seed Changes and Polyembryony During the Seed Development Process of P. bournei

This study measured the seed characteristics (thousand grain weight, length, width, perimeter, area, etc.) of 13 plus trees (Table 2). The seed with the largest weight per thousand was No. 12, with a width of up to 7.62 ± 0.05 mm and an area of up to 66.70 ± 0.35 mm^2^; clone No. 13 showed a seed length of 14.04 ± 0.04 mm, and the longest perimeter was 33.61 ± 0.12 mm; the seed No. 8 had the smallest length, width, and perimeter, with a length of only 10.98 ± 0.05 mm, a width of only 5.52 ± 0.05 mm, and was the smallest in morphology. In terms of thousand grain weight, No. 12 attained 346.50 ± 0.05 g, significantly exceeding all other accessions (*p* < 0.01); for seed dimension traits, No. 7 had the greatest seed length of 14.69 ± 0.98 mm, which differed statistically from No. 8 (*p* < 0.01); No. 12 bore the widest seeds (7.62 ± 0.05 mm), also significantly larger than No. 8 (*p* < 0.01). Regarding seed perimeter, No. 13 (33.61 ± 0.12 mm) and No. 7 (33.13 ± 0.09 mm) showed substantially larger values than the remaining genotypes (*p* < 0.01), whereas no statistical divergence was detected between these two clones. For seed area, No. 12 (66.70 ± 0.35 mm^2^) was significantly superior to all other materials (*p* < 0.01). This indicates that the seeds of *P. bournei* from different plus trees have significant differences in thousand grain weight, length, perimeter, and area.

**Figure 3:**
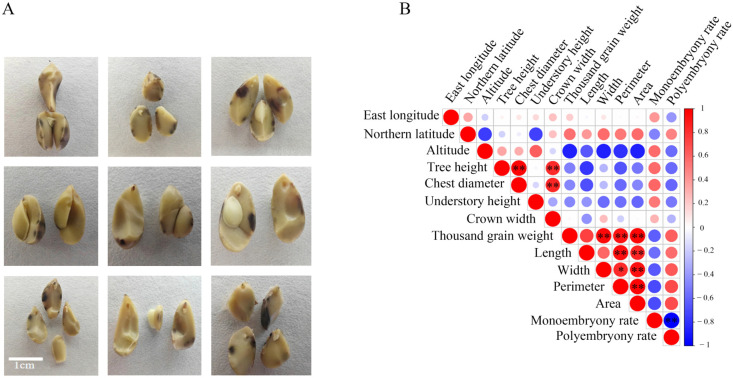
Polyembryonic seed morphology and correlation with maternal and environmental variables in *P. bournei*. (**A**) Morphological characteristics of *P. bournei* polyembryonic seeds. Note: The above are all various forms of polyembryonic seeds (two embryos and three embryos are not distinguished); (**B**) Correlation analysis of polyembryonic seeds of *P. bournei* seeds and environmental factors of the harvesting site. Red and blue indicate positive and negative correlations, respectively, * *p* < 0.05, ** *p* < 0.01.

Subsequently, seed coats were manually stripped to observe the internal structure of *P. bournei* seeds (Figure 3A). Cotyledons from monoembryonic seeds regularly split into two symmetrical lobes, whereas those of polyembryonic seeds commonly separated into three, four or more irregularly shaped segments. Rapid oxidative browning with black speck formation frequently occurred during seed coat removal and seed dissection. The anatomical statistical analysis on seeds from the 13 plus trees showed that the monoembryony rate was consistently higher than the polyembryony rate across all plus trees, with significant inter-tree variation in polyembryony rate (Table 2). Among them, polyembryony seed counts and rates for accessions No. 4 and No. 13 were markedly greater than those of No. 6, No. 7 and No. 9. In particular, No. 13 achieved a polyembryony rate of 30.00 ± 0.02%, differing significantly from all remaining plus-tree progenies (*p* < 0.01).

Correlation analysis (Figure 3B) revealed that geographic variables (longitude, latitude, and altitude indicators of the plus trees) had no significant correlation with seed morphology traits or thousand grain weight, which is likely attributed to the narrow geographic span and limited environmental gradients of sampling locations in southern Fujian; accordingly, potential impacts of broader geographic and altitudinal variation on seed traits cannot be excluded. The thousand grain weight was extremely significantly and positively correlated with seed width, perimeter and area (*p* < 0.01). Seed width was also positively correlated with thousand-kernel weight at the same highly significant level (*p* < 0.01). Seed perimeter and area were significantly associated with seed length and width (*p* < 0.05), while perimeter showed an extremely significant positive correlation with seed area (*p* < 0.01). By contrast, polyembryony rate had no significant correlation with any of the tested seed or environmental indicators. In summary, within the current sampling range, seed thousand grain weight was strongly positively correlated with seed width, perimeter and area (*p* < 0.01), whereas polyembryony rate was independent of all measured environmental and morphological parameters. These findings are limited to the present sampling conditions and measured indicators, and cannot exclude the potential effects of other unmonitored environmental factors or broad environmental gradients on polyembryony occurrence. Since no significant association was observed between polyembryony rate and the measured variables, genetic and molecular regulation are presumed to dominate polyembryony formation, and the underlying mechanisms require further exploration.

### 3.2. Observation on Rooting of P. bournei Seeds

**Figure 4:**
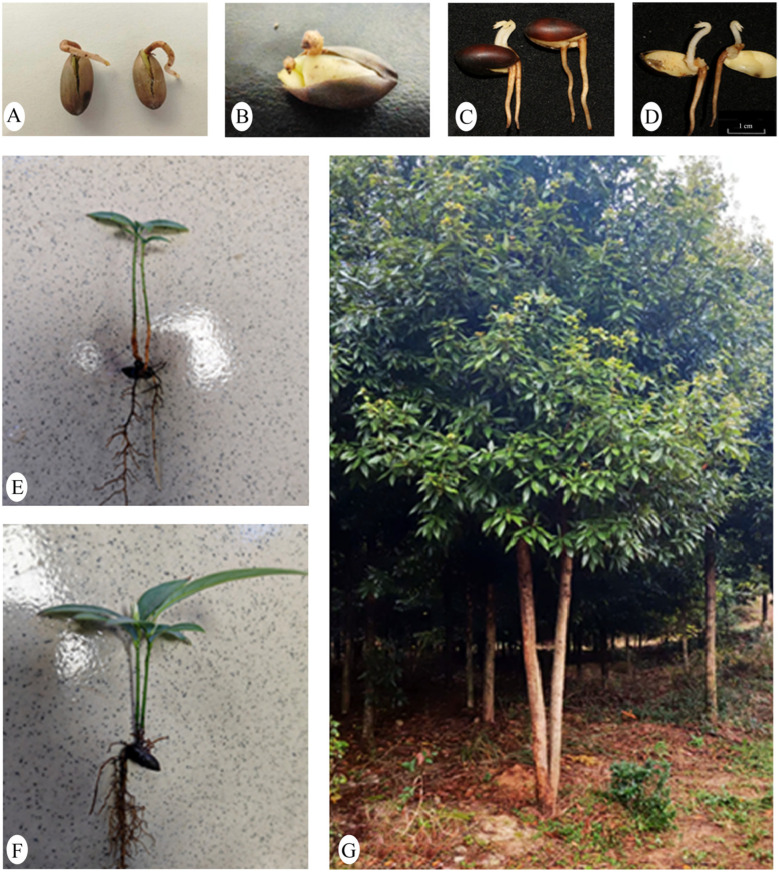
Observation of root formation of monoembryonic and polyembryonic seeds of *P. bournei* and the growth condition of polyembryonic plants. (**A**) Rooting condition of monoembryonic seeds; (**B**) Rooting condition of polyembryonic seeds; (**C**) Rooting and budding of polyembryonic seeds; (**D**) Separation morphological characteristics of a polyembryonic seed; (**E**) Size-dimorphic plants; (**F**) Twin plants; (**G**) Polyembryonic trees in *P. bournei*.

By observing the morphological changes during the germination process of *P. bournei* seeds (Figure 4), we found that the radicle usually breaks through the seed coat from the top of the seed (Figure 4A). Polyembryonic seeds exhibited obvious variation in radicle emergence sites: radicles may emerge solely from the apex, or simultaneously from the apex and the middle part of the seed (Figure 4B). This phenomenon directly reflects the specificity of hypocotyl and radicle differentiation during the development of polyembryonic seeds. Each embryo within polyembryonic seeds can independently develop into intact radicle and embryo structures (Figure 4C). After breaking the seed open, it can be seen that two roots and two buds coexist (Figure 4D).

Qualitative observations based on morphological characteristics showed that seedlings germinated from polyembryonic seeds of *P. bournei* could be divided into two main forms: size-dimorphic plants and twin plants. In the size-dimorphic plants’ morphology (Figure 4E), although both young plants can develop, there is a significant difference in growth. One plant has normal stem and leaves, and a good growth rate; the other plant has a thin stem and small leaves, and a weaker growth rate. It is speculated that such differences may result from uneven resource allocation and developmental competition among embryos within seeds. In the twin plants (Figure 4F), the two seedlings were similar in size and growth, with relatively balanced overall development. This indicates weak competition among embryos or relatively balanced resource utilization in this morphotype, which may confer potential advantages in growth stability. In addition, our research group discovered tall and similar-growing adult *P. bournei* plants in Hunan Province during a field plant survey (Figure 4G), Based on this, it is speculated that they may be derived from bimonoecious polyembryonic seedlings, providing preliminary field evidence suggesting that the polyembryonic seedlings have the potential to develop into healthy adult plants.

### 3.3. Comparative Transcriptome Analysis of Monoembryonic and Polyembryonic Seeds of P. bournei

RNA-seq analysis was performed on monoembryonic seeds and polyembryonic seeds of *P. bournei*. After raw data filtering, each sample yielded 6.09–6.26 Gb of effective base pairs, with an error rate of 0.03, and the Q20 base percentages were all above 96.45%, the Q30 value was above 92.84%, and the GC content was between 45.58 and 45.96%, indicating that the sequencing quality was higher (Table S3). The clean reads obtained were compared with the reference genome of *P. bournei*, and the overall comparison efficiency was between 87.51% and 91.83%, and the percentage of reads in each sample that mapped to the genome was greater than 87.51% (Table S4). These results confirmed that the RNA-seq data had high quality and were suitable for further analysis.

**Figure 5:**
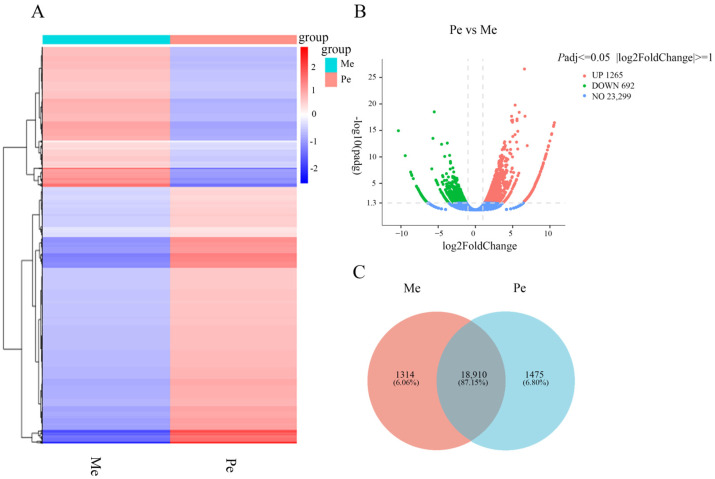
Analysis of DEGs in monoembryonic and polyembryonic morphology of *P. bournei*. (**A**) Clustering heatmap of DEGs between monoembryony (Me) and polyembryony (Pe). (**B**) Volcano plot of DEGs between Me and Pe. Dashed lines indicate thresholds: *p*_adj_ = 0.05 (horizontal) and |log_2_FC| = 1 (vertical). (**C**) Venn diagram of co-expression genes between Me and Pe.

Cluster analysis was performed on DEGs derived from monoembryonic and polyembryonic ovule samples (Figure 5A). Genes were clustered into distinct expression modules with divergent transcription patterns between the two phenotypes. Differential expression analysis was implemented via DESeq2, with DEGs filtered by thresholds of |log_2_ Fold Change| > 1 and adjusted *p*_adj_ < 0.05, In total, 1957 DEGs were screened out, consisting of 1265 upregulated and 692 downregulated transcripts (Figure 5B). Venn diagram analysis (Figure 5C) illustrated the distribution of all expressed genes (FPKM > 0) in monoembryonic and polyembryonic seeds. A total of 1314 genes were uniquely expressed in monoembryonic seed samples, and 1475 genes were specifically expressed in polyembryonic seed samples. The two groups shared 18,910 co-expressed genes, accounting for 87.15% of all expressed genes, while differentially expressed genes only accounted for 10.35% of the co-expressed genes. These results revealed that gene expression profiles were highly conserved between monoembryonic and polyembryonic seeds. The vast majority of genes were stably expressed in both types of seeds, and only a small number of differentially expressed genes participated in the regulatory processes related to polyembryony development.

### 3.4. Functional Annotation Analysis of DEGs

**Figure 6:**
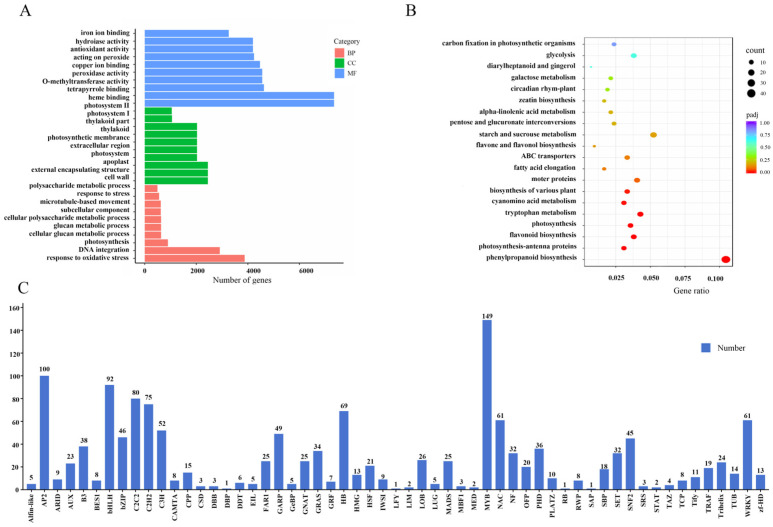
Functional enrichment and transcription factor analysis of DEGs between monoembryonic and polyembryonic *P. bournei*. (**A**) GO enrichment map of DEGs between monoembryony and polyembryony (BP: biological process; CC: cellular component; MF: molecular function); (**B**) KEGG pathway enrichment analysis map of DEGs between monoembryony and polyembryony; (**C**) Statistics of transcription factor family members identified from all expressed genes of monoembryonic and polyembryonic *P. bournei* seeds.

The GO functional enrichment information of the selected DEGs was analyzed. The top 30 significantly enriched entries are shown in the figure (Figure 6A). Genes upregulated in polyembryonic seeds of *P. bournei* exhibited significant enrichment across biological process (BP), molecular function (MF) and cellular component (CC), indicating that polyembryo development involves functional remodeling at multiple levels. Among them, the three most enriched items were photosystem II (GO:0009523), heme binding (GO:0020037), and tetrapyrrole binding (GO:0046906), the latter two belong to the MF category, while photosystem II belongs to CC. These terms collectively indicate enhanced functions related to energy capture and electron transport in polyembryonic seeds. By increasing the activity of photosynthesis-related complexes and the binding capacity of oxidoreductase cofactors, these processes provide sufficient energy and substances for the synchronous development of multiple embryos, thereby ensuring the stable formation of polyembryonic structures.

KEGG pathway analysis was performed on the DEGs, and the 20 most significantly enriched pathways with the smallest FDR values were selected and plotted as a bubble graph (Figure 6B). The results showed that the majority of the DEGs were enriched in phenylpropanoid biosynthesis (ath00940), and there was partial enrichment in tryptophan metabolism (ath00380) and starch and sucrose metabolism (ath00500). Activation of the phenylpropanoid biosynthesis pathway accelerates the production of cell wall precursors and flavonoids, modulates embryonic cell division and differentiation, and provides structural support for the initiation of polyembryonic primordia. The enriched tryptophan metabolism pathway is tightly linked to auxin biosynthesis, which governs embryonic polarity establishment and growth balance, and ultimately affects polyembryo morphogenesis. Upregulated starch and sucrose metabolism elevates the transport and utilization efficiency of carbon sources, continuously delivers energy and nutrients for polyembryo development, and maintains the coordinated growth of multiple embryos.

Transcription factors (TFs) can bind to specific gene regulatory regions, activate or inhibit gene expression, and then regulate cell differentiation, metabolic pathways or signaling pathways, ultimately driving phenotypic changes. In this study, transcription factor annotation was performed based on all expressed genes from monoembryonic and polyembryonic seeds of *P. bournei*. A total of 1464 TFs distributed across 57 transcription factor families were identified (Figure 6C). Among them, the *MYB* (149, 10.2%) and *AP2* (100, 6.83%) gene families had a higher number of annotated genes, followed by *bHLH* (92, 6.28%), *C2C2* (80, 5.46%), and *C2H2* (75, 5.12%). Additionally, eight members of the RWP gene family were also annotated. RWP is an important transcription factor in plants, playing a crucial role in the gametophyte development and embryo formation processes of plants. Therefore, the RWP gene family was further analyzed based on the whole genome data of *P. bournei*.

### 3.5. Identification and Physicochemical Properties of the RWP Gene Family in P. bournei

A total of eight RWP gene family members were identified in the whole genome data of *P. bournei*. According to their sequence numbers, they were named *PbRWP1*–*PbRWP8*. The proteins encoded by the RWP family genes contain 201 to 1164 amino acids, with a relative molecular weight of 23.00 to 128.32 kDa and a theoretical isoelectric point (pI) of 5.30 to 9.80. It is worth noting that proteins with an isoelectric point above 7 are basic proteins, while those with an isoelectric point below 7 are acidic proteins. Among them, *PbRWP2* and *PbRWP5* are basic proteins, while the rest are acidic proteins. The instability index was 48.08 to 59.04, and the aliphatic index was 66.57 to 83.98. The protein hydrophilicity was all less than 0, indicating that all proteins are hydrophilic proteins (Table 3).

**Figure 7:**
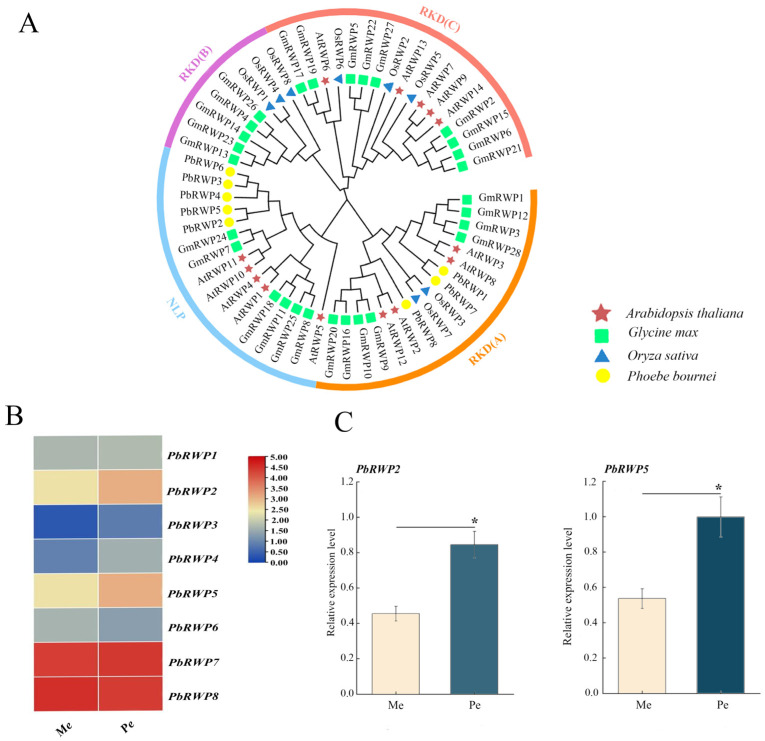
Phylogeny and expression pattern analysis of the RWP gene family. (**A**) Phylogenetic tree analysis of RWP gene family; (**B**) Gene expression heatmap; (**C**) qRT-PCR verification, * *p* < 0.05.

Based on the NJ method, a phylogenetic relationship was constructed for a total of 58 RWP protein sequences from *P. bournei*, *Arabidopsis thaliana*, *Oryza sativa*, and *Glycine max* (Figure 7A). According to the classification of the *Arabidopsis thaliana AtRWP* family and topological structure, all RWP proteins were classified into two-branch structure: *NLP* class (16) and the *RKD* class (42), and the *RKD* branch was further divided into three subcategories: A (17), B (8) and C (17). The eight RWP genes of *P. bournei* were clustered in the *NLP* class and *RKD* (A) class, with *PbRWP1*, *PbRWP7*, and *PbRWP8* clustering in the *RKD* (A) class and the remaining genes clustering in the *NLP* class. This distribution pattern is consistent with the evolutionary differentiation of the RWP family in some dicotyledonous species.

Based on FPKM-normalized expression data from RNA-seq, a heatmap (Figure 7B) was constructed to illustrate the expression profiles of the *PbRWP* gene family in monoembryonic and polyembryonic tissues. The results showed that *PbRWP7* and *PbRWP8* were highly expressed in both monoembryony and polyembryony, and the expression levels in polyembryony were slightly higher than those in monoembryony, with slightly higher expression levels in polyembryonic tissues, and no statistically significant difference was detected between the two groups. *PbRWP3* exhibited low expression in both embryonic tissues. *PbRWP1* and *PbRWP6* showed stable expression levels across the two tissue types and had no obvious tissue-specific expression characteristics. The transcript abundances of *PbRWP2* and *PbRWP5* were relatively low in monoembryonic tissues but markedly upregulated in polyembryonic tissues, and this distinct expression difference was clearly visualized in the heatmap. Collectively, we propose that *PbRWP2* and *PbRWP5* are key candidate genes regulating polyembryony development in *P. bournei*.

To verify the reliability of the transcriptome data and clarify the actual expression differences of *PbRWP2* and *PbRWP5*, qRT-PCR was performed in this study (Figure 7C). The results demonstrated that the relative expression levels of *PbRWP2* and *PbRWP5* were significantly higher in polyembryonic tissues than in monoembryonic tissues (*p* < 0.05), which was consistent with the FPKM-based expression patterns obtained from transcriptome analysis.

## 4. Discussion

Polyembryony, a phenomenon whereby multiple embryos exist in a single seed, has been documented in *Arabidopsis thaliana* [27], *Mangifera indica* [28], and *Zea mays* [29]. From an evolutionary perspective, polyembryony enables one seed to germinate multiple seedlings and elevates the probability of successful seedling establishment, functioning as an adaptive reproductive compensation strategy for maternal trees. Conversely, resource competition among sibling embryos within a constrained seed frequently triggers aberrant embryo and seedling development, impairing seedling survival [30]. In our present experiment, two morphotypes of polyembryonic seedlings were observed in *P. bournei*: size-dimorphic seedlings and twin seedlings with uniform growth. Furthermore, our field inventory across Hunan Province identified mature paired twin *P. bournei* trees with comparable stem diameter and growth status. This field evidence suggests that polyembryonic seedlings of *P. bournei* are able to develop into vigorous mature individuals under favorable environmental and cultivation regimes and retain robust capacity for wild colonization and sustainable growth.

Researchers believe that there may be a certain correlation between the probability of multiple embryos occurring, the seed size and the growth environment of the plant. Studies have shown that the degree of polyembryony in citrus varieties is influenced by environmental factors, and the polyembryos vary between different seeds, fruits, plant tissues, and years [31]. Long-term high-temperature stress will cause abnormal proliferation of rape embryo suspensor cells to form secondary embryos, reflecting the impact of temperature, an external environmental factor, on plant embryo development and polyembryo-related phenomena [32]; Sidhu et al. reported that the embryo rate was positively correlated with the average embryo number, seed quality, height, and width, further underscoring the potential link between seed morphological characteristics and polyembryonic occurrence in certain plant species [33]. Distinct from annual crops and evergreen fruit trees, the present study adopted *P. bournei*, a perennial precious broad-leaved tree species, as the research material. Correlation analysis showed that within the sampling gradient, environmental factors including longitude, latitude and altitude, as well as seed morphological traits, had no significant correlation with the polyembryony rate of *P. bournei*. Notably, only several macro-environmental indicators were examined in this study, while micro-environmental factors such as light, soil, temperature and humidity were not included. The absence of dominant effects of the detected factors does not rule out their potential roles in embryonic development. In addition, correlation analysis merely demonstrates variable correlations, yet it is unable to reveal causality. Based on current findings, we preliminarily conclude that the polyembryonic trait of *P. bournei* is mainly regulated by endogenous genetic and molecular pathways. Further functional experiments are required to elucidate the underlying causal mechanisms.

The mechanism of polyembryony is complex, and one of the main reasons may be apomixis. Apomixis is a reproductive method that bypasses meiosis and the fusion of male and female gametes. It reproduces offspring through asexual embryos or asexual seeds. The offspring it produces are genetically identical to the parent plant [34]. Apomixis is common in both herbaceous and woody plants, such as *Paspalum* [35], *Herba taraxaci* [34], *Hawkweed* [36], *Zanthoxylum bungeanum* [37] and *Citrus* [38]. On the surface, apomixis seems to be contrary to the principles of biological diversity, but in fact, only a few plants exhibit the characteristics of obligate apomixis. Apomixis provides plants with a unique mechanism to maintain and restore high-quality hybrid gene combinations [39]. For instance, Wang C et al. established an apomictic reproductive system through gene editing technology for hybrid rice, and successfully achieved stable inheritance of hybrid rice genotypes [40]. In addition, researchers can also obtain parthenogenetic lettuce materials by transferring genes that control apomixis in dandelion into lettuce [41]. Research by Wang X et al. has shown that apomixis is also a significant feature of modern citrus, with the *CitRWP* gene showing higher expression levels in ovules of polyembryonic citrus cultivars [42]. Furthermore, apomixis is commonly observed in *Zanthoxylum* plants, and the research of Hu L et al. further revealed the potential role of the *ZaRWP* gene in apomixis of *Zanthoxylum armatum* [43]. Based on published studies of homologous woody species, polyembryony formation in *P. bournei* is presumed to be correlated with the apomixis pathway. Given the lack of supporting embryological anatomical evidence, the definite causal link between the two biological processes still needs to be validated by further embryological tests. Relevant follow-up research will lay a critical foundation for elite variety selection and clonal breeding of *P. bournei*.

The plant RWP transcription factors are key regulators of gametophyte development and play an important regulatory role in early embryo morphogenesis and plant nitrogen utilization [44,45]. Multiple studies have demonstrated that members of the RWP gene family exhibit a conserved pattern of transition from quantitative expression changes to qualitative phenotypic alterations in regulating plant polyembryony development. In woody plant research, Wang et al. [42] found that the *CitRWP* gene is specifically and highly expressed in the ovules of polyembryonic citrus cultivars, confirming that its expression level is closely associated with the polyembryonic trait. Using *Fortunella hindsii* as the research material, Song et al. [19] verified that *FhRWP* can induce the formation of somatic embryogenic calli and reactivate the cell fate of somatic embryos. Variations in the promoter of mango *MiRWP* can upregulate gene expression and thereby trigger nucellar embryo formation. This further indicates that the expression level of RWP genes directly governs the polyembryonic phenotype of woody plants [17,28]. Related studies on reproductive development in *Zanthoxylum armatum* have also confirmed that the RWP-RK family serves as a core regulatory target for apomictic embryogenesis in woody plants [16,46], which supports the high conservation of the RWP-mediated somatic embryogenesis pathway in perennial woody tree species. Asih et al. [47] also verified, in herbaceous plants, that *OsRKD3* from black rice can activate the somatic embryogenesis program, further supporting that the functions of this gene family in regulating embryonic development are widespread across the plant kingdom. In the present study, eight RWP family members were identified in *P. bournei*, among which *PbRWP2* and *PbRWP5* exhibited pronounced expression differences between monoembryony and polyembryony, and the expression levels were significantly upregulated in polyembryonic seeds. The results of qRT-PCR analysis were consistent with the trend of transcriptome data. Combining the transcriptome and qRT-PCR expression results obtained in this study, we preliminarily identified *PbRWP2* and *PbRWP5* as key candidate genes involved in polyembryony development of *P. bournei*. Nevertheless, this study only revealed a correlation between their expression and the polyembryonic phenotype, and relevant evidence for gene functional verification is still lacking. The specific regulatory pathways and causal mechanisms of these two genes remain to be elucidated in further experiments.

## 5. Conclusions

This study systematically explored the inducing factors and potential regulatory mechanisms underlying polyembryony in *P. bournei* seeds from three perspectives: macroscopic environmental factors, seed phenotypic traits, and transcriptome analysis. Correlation analysis showed that the latitude, longitude and altitude of sampling sites, as well as seed morphological indices, had no significant correlation with the polyembryony rate of *P. bournei*. Comparative transcriptome analysis showed that DEGs were significantly enriched in the two processes of phenylpropanoid biosynthesis and sucrose and starch metabolism, and RWP transcription factors may be involved in polyembryonic development in *P. bournei*. A total of eight members were identified in the RWP gene family of *P. bournei* and were classified into two classes according to conserved domains, named *NLP* and *RKD* (A). Both transcriptomic data and qRT-PCR validation confirmed that *PbRWP2* and *PbRWP5* were significantly upregulated in polyembryonic seeds, suggesting the key roles in regulating polyembryony. In conclusion, the research preliminarily explored the potential factors of polyembryony formation in *P. bournei*, laying a theoretical foundation for further polyembryonic studies.

## Figures and Tables

**Table 1:** Characteristics and locations of 13 *P. bournei* plus trees.

Number	Collection Location	East Longitude/E	North Latitude/N	Altitude/m	Tree Height/m	Chest Diameter/cm	Understory Height/m	Crown Width/m
1	Kangfu, Yangkou, Shunchang	117.51	26.44	173.4	21	46.1	7.0	8 × 12
2	Tiantai Mountain, Yangkou, Shunchang	117.51	26.42	543.3	22	69.7	6.5	7.5 × 11
3	Yongji, Guangming, Jiangle	117.25	26.46	206.4	19	48.8	4.6	8 × 11
4	Yongji, Guangming, Jiangle	117.25	26.46	216.7	22	52.8	2.3	8 × 9.5
5	Gekeng, Guangming, Jiangle	117.24	26.46	242.6	23	50.6	6.5	6 × 9
6	Baishakeng, Yangkou, Shunchang	117.53	26.44	259.9	21	37.3	9.0	6 × 8
7	Xiejian, Yangkou, Shunchang	117.54	26.48	138.0	15	34.8	4.7	7 × 8
8	Xibian, Jianxi, Shunchang	117.55	26.51	163.5	19	53.5	3.0	8 × 10
9	Nanshan, Jianxi, Shunchang	117.56	26.53	248.1	33	135.6	3.1	16 × 20
10	Xialinchang, Jianxi, Shunchang	117.55	26.53	304.6	16	26.1	7.5	4 × 6
11	Anxia, Jianxi, Shunchang	117.57	26.49	133.8	25	56.0	3.7	8 × 10
12	Hengkeng, Jianxi, Shunchang	117.57	26.49	158.8	22	59.2	7.0	16 × 18
13	Xiaohu, Jianxi, Shunchang	117.54	26.53	188.4	14	25.3	3.5	6 × 8

**Table 2:** Analysis of seed traits and polyembryony rate of 13 plus trees of *P. bournei*.

Number	Thousand Grain Weight/g	Seed Size				Monoembryony		Polyembryony	
		Length/mm	Width/mm	Perimeter/mm	Area/mm^2^	Number	Embryo Rate/%	Number	Embryo Rate/%
1	199.68 ± 2.23 FG	11.49 ± 0.08 DE	5.61 ± 0.01 DE	28.14 ± 0.16 DEF	46.67 ± 0.28 EF	46.00 ± 0.53 AB	92.00 ± 0.01 AB	4.00 ± 0.53 DEF	8.00 ± 0.01 DEF
2	204.85 ± 0.60 FG	11.68 ± 0.02 DE	5.60 ± 0.03 DE	28.42 ± 0.07 DEF	47.71 ± 0.31 EF	46.00 ± 1.00 AB	92.00 ± 0.02 AB	4.00 ± 0.56 DEF	8.00 ± 0.01 DEF
3	217.43 ± 3.10 E	13.03 ± 0.12 BC	6.06 ± 0.07 CDE	31.35 ± 0.29 B	57.29 ± 1.20 BC	44.00 ± 0.56 AB	88.00 ± 0.01 AB	6.00 ± 1.05 CDE	12.00 ± 0.02 CDE
4	217.36 ± 3.28 E	12.41 ± 0.05 CD	6.19 ± 0.05 BCDE	30.47 ± 0.12 BC	56.08 ± 0.57 CD	36.00 ± 0.78 CD	72.00 ± 0.01 CD	14.00 ± 1.23 AB	28.00 ± 0.02 AB
5	195.46 ± 3.14 G	11.08 ± 0.09 E	5.59 ± 0.04 DE	27.73 ± 0.54 EF	46.21 ± 1.41 F	40.00 ± 1.53 BCD	80.00 ± 0.03 BCD	10.00 ± 1.26 BC	20.00 ± 0.03 BC
6	207.34 ± 0.71 EF	12.01 ± 0.05 CDE	5.60 ± 0.03 DE	29.00 ± 0.16 CDE	49.30 ± 0.36 DEF	47.00 ± 1.16 A	94.00 ± 0.02 A	3.00 ± 0.38 EF	6.00 ± 0.01 EF
7	303.48 ± 3.09 C	14.69 ± 0.98 A	6.30 ± 0.03 BCD	33.13 ± 0.09 A	64.14 ± 0.49 AB	47.00 ± 1.47 A	94.00 ± 0.03 A	3.00 ± 0.83 EF	6.00 ± 0.02 EF
8	179.03 ± 3.31 H	10.98 ± 0.05 E	5.52 ± 0.05 E	27.21 ± 0.23 F	44.40 ± 0.41 F	44.00 ± 1.39 AB	88.00 ± 0.03 AB	6.00 ± 0.51 CDE	12.00 ± 0.01 CDE
9	209.88 ± 4.76 EF	11.91 ± 0.03 CDE	6.03 ± 0.07 CDE	29.64 ± 0.31 CD	53.75 ± 0.58 CDE	48.00 ± 2.08 A	96.00 ± 0.04 A	2.00 ± 0.67 F	4.00 ± 0.01 F
10	208.46 ± 1.27 EF	12.06 ± 0.05 CDE	5.65 ± 0.03 CDE	28.98 ± 0.13 CDE	49.56 ± 0.51 DEF	42.00 ± 1.73 ABC	84.00 ± 0.03 ABC	8.00 ± 1.13 CD	16.00 ± 0.02 CD
11	329.50 ± 2.83 B	11.72 ± 0.08 DE	6.80 ± 0.59 B	30.36 ± 0.99 BC	58.94 ± 5.76 BC	43.00 ± 0.52 AB	86.00 ± 0.01 AB	7.00 ± 1.19 CDE	14.00 ± 0.02 CDE
12	346.50 ± 0.05 A	11.71 ± 0.04 DE	7.62 ± 0.05 A	31.64 ± 0.04 B	66.70 ± 0.35 A	40.00 ± 1.35 BCD	80.00 ± 0.05 BCD	10.00 ± 1.26 BC	20.00 ± 0.02 BC
13	288.80 ± 2.61 D	14.04 ± 0.04 AB	6.38 ± 0.04 BC	33.61 ± 0.12 A	64.17 ± 0.46 AB	35.00 ± 1.50 D	70.00 ± 0.03 D	15.00 ± 1.14 A	30.00 ± 0.02 A

Note: Different capital letters in the table indicate highly significant differences between different plus tree seeds (*p* < 0.01).

**Table 3:** Basic information of *RWP* gene family in *P. bournei*.

Sequence ID	Gene Name	Number of Amino Acids	Molecular Weight (kDa)	Isoelectric Point	Instability Index	Aliphatic Index	Hydrophilicity Index
Maker00048578	*PbRWP1*	1032	114.19	5.45	59.04	78.03	−0.385
Maker00025725	*PbRWP2*	906	101.10	7.88	48.08	76.94	−0.421
Maker00044782	*PbRWP3*	1164	128.32	6.31	56.05	77.35	−0.401
Maker00007515	*PbRWP4*	956	106.47	5.99	51.60	77.44	−0.389
Maker00029939	*PbRWP5*	201	23.00	9.80	55.93	66.57	−0.864
Maker00044800	*PbRWP6*	694	76.64	5.69	48.09	83.98	−0.234
Maker00034339	*PbRWP7*	1037	113.35	5.30	57.61	76.34	−0.311
Maker00019978	*PbRWP8*	945	103.93	5.79	48.81	75.39	−0.367

## Data Availability

Statistical analyses are included in the Supplementary Materials. Raw transcriptome data have been deposited in the NCBI Sequence Read Archive (SRA) under the BioProject accession PRJNA1463751 (https://dataview.ncbi.nlm.nih.gov/object/PRJNA1463751)/(https://dataview.ncbi.nlm.nih.gov/object/PRJNA1463751?reviewer=f6eqd7nlgdeopoor9nva6p2ufl/ accessed on 8 May 2026), with associated BioSample accessions SAMN59600004–SAMN59600009. All data will be made publicly available upon publication of this article.

## Supplementary Materials

The following supporting information can be downloaded at https://www.mdpi.com/article/10.3390/biology15120919/s1, Table S1: The PCR reaction system and procedure; Table S2: Primer sequences; Table S3: Monoembryonic and polyembryonic sample sequencing data quality statistics table; Table S4: The comparison results of monoembryonic and polyembryonic samples with reference genomes.

## Abbreviations

The following abbreviations are used in this manuscript:

IAA	Indole-3-acetic acid
IBA	Indole-3-butyric acid
SA	Salicylic acid
qRT-PCR	Quantitative reverse transcription polymerase chain reaction
NGDC	National Genomics Data Center
FPKM	Fragments Per Kilobase of transcript per Million mapped reads
DEGs	Differentially expressed genes
GO	Gene Ontology
KEGG	Kyoto Encyclopedia of Genes and Genomes
CDD	Conserved Domain Database
NCBI	National Center for Biotechnology Information
NJ	Neighbor-joining
BP	Biological processes
MF	Molecular function
CC	Cellular component
TFs	Transcription factors
