# Supplementary material for: Transcriptomic Analysis and Physiological Indicators Synthetically Uncover Candidate Genes Regulating Polyembryony Formation of Phoebe bournei Seeds
Source: Biology (Basel). 2026 Jun 12;15(12):919. doi: 10.3390/biology15120919 (PMC13295622; doi:10.3390/biology15120919)
Supplement: Supplementary file 1 [file biology-15-00919-s001.zip › biology-4345040-supplementary.pdf]

Supplementary Material

Table S1. The PCR reaction system and procedure

Reagent	Usage amount	Reaction conditions
2xTaq Pro Universal SYBR Qpcr Master Mix	10.0 μL	95℃ pre-denaturation 30 s
Primer1(10 μM)	0.4 μL	95℃ denaturation 10 s
Primer2(10 μM)	0.4 μL	60℃ annealing 34 s
Template DNA/cDNA	0.2 μL	95℃ 15 s to 60℃ 1 min<br>Then 95℃ 15 s
dd H2O	20 μL	Store at 4℃

Table S2. Primer sequences

Gene name	Forward primer sequence (5'-3')	Reverse primer sequence (5'-3')
PbRWP2	CTCAACCTCCTCATCCACAAC	GCAGAAGCAGAAGCAGAAGT
PbRWP5	ATCCTCCTCCATCATCGTCTC	GCAGAAGCAGAAGCAGAAGT

Table S3. Monoembryonic and polyembryonic sample sequencing data quality statistics table

Sample	Raw reads (M)	Clean reads (M)	Clean bases (G)	Error rate	Clean reads Q20	Clean reads Q30	GC percent (%)
Me-1	44.12	41.60	6.24	0.03	97.88	94.23	45.58
Me-2	43.71	41.80	6.26	0.03	98.32	93.89	45.96
Me-3	44.37	42.74	6.18	0.03	97.65	93.25	45.75
Pe-1	42.31	40.66	6.09	0.03	97.54	93.56	45.63
Pe-2	43.71	40.97	6.15	0.03	97.78	93.38	45.81
Pe-3	45.39	41.37	6.21	0.03	96.45	92.84	45.93

Table S4. The comparison results of monoembryonic and polyembryonic samples with reference genomes

Sample	Total reads (M)	Reference genome alignment results total mapping (%)	Reference genome alignment results uniquely mapping (%)
Me-1	41.60	91.01	88.03
Me-2	41.38	91.51	87.84
Me-3	42.84	91.83	87.51
Pe-1	40.56	87.63	84.74
Pe-2	40.66	87.51	84.83
Pe-3	41.34	87.93	85.16
